# TLR Tolerance Reduces IFN-Alpha Production Despite Plasmacytoid Dendritic Cell Expansion and Anti-Nuclear Antibodies in NZB Bicongenic Mice

**DOI:** 10.1371/journal.pone.0036761

**Published:** 2012-05-04

**Authors:** Evelyn Pau, Yui-Ho Cheung, Christina Loh, Ginette Lajoie, Joan E. Wither

**Affiliations:** 1 Arthritis Centre of Excellence, Toronto Western Research Institute, Toronto, Ontario, Canada; 2 Department of Immunology, University of Toronto, Toronto, Ontario, Canada; 3 Department of Pathology, Mount Sinai Hospital and William Osler Health Centre, Toronto, Ontario, Canada; 4 Department of Medicine, University Health Network, Toronto, Ontario, Canada; University Paris Sud, France

## Abstract

Genetic loci on New Zealand Black (NZB) chromosomes 1 and 13 play a significant role in the development of lupus-like autoimmune disease. We have previously shown that C57BL/6 (B6) congenic mice with homozygous NZB chromosome 1 (B6.NZBc1) or 13 (B6.NZBc13) intervals develop anti-nuclear antibodies and mild glomerulonephritis (GN), together with increased T and B cell activation. Here, we produced B6.NZBc1c13 bicongenic mice with both intervals, and demonstrate several novel phenotypes including: marked plasmacytoid and myeloid dendritic cell expansion, and elevated IgA production. Despite these changes, only minor increases in anti-nuclear antibody production were seen, and the severity of GN was reduced as compared to B6.NZBc1 mice. Although bicongenic mice had increased levels of *baff* and *tnf-α* mRNA in their spleens, the levels of IFN-α-induced gene expression were reduced. Splenocytes from bicongenic mice also demonstrated reduced secretion of IFN-α following TLR stimulation *in vitro*. This reduction was not due to inhibition by TNF-α and IL-10, or regulation by other cellular populations. Because pDC in bicongenic mice are chronically exposed to nuclear antigen-containing immune complexes *in vivo*, we examined whether repeated stimulation of mouse pDC with TLR ligands leads to impaired IFN-α production, a phenomenon termed TLR tolerance. Bone marrow pDC from both B6 and bicongenic mice demonstrated markedly inhibited secretion of IFN-α following repeated stimulation with a TLR9 ligand. Our findings suggest that the expansion of pDC and production of anti-nuclear antibodies need not be associated with increased IFN-α production and severe kidney disease, revealing additional complexity in the regulation of autoimmunity in systemic lupus erythematosus.

## Introduction

Systemic lupus erythematosus (SLE) is a multisystem autoimmune disease of unknown etiology. One of the hallmarks of this condition is the loss of tolerance to self-antigens, particularly nuclear Ag, leading to production of anti-nuclear antibodies (ANA) and formation of immune complexes (IC) [1]. Deposition of these IC in the glomeruli, skin, joints, and other organs induces tissue damage resulting in the manifestations of disease including; glomerulonephritis (GN), skin rash, and arthritis [2].

The New Zealand Black (NZB) mouse and its F_1_ cross with the New Zealand White mouse (NZB/W) closely mimic SLE and characterization of the immune defects in these mice has led to a number of fundamental insights into the human disease [3]. To facilitate identification of NZB lupus susceptibility loci, we have produced congenic mouse strains, in which homozygous NZB chromosomal intervals containing a single or cluster of susceptibility alleles have been introgressed onto the non-autoimmune C57BL/6 (B6) background. We have previously shown that congenic mice with NZB chromosome 1 (B6.NZBc1) or chromosome 13 (B6.NZBc13) intervals develop ANA and mild GN [4]–[5]. In both mouse strains, this was accompanied by several cellular phenotypic abnormalities including splenomegaly and increased proportions of activated T and B cells; however, the nature of the autoantibody (autoAb) produced and immune defects in these mice differed [5]–[6]. B6.NZBc1 mice demonstrate a breach of tolerance to nuclear antigens that is characterized by the presence of histone-reactive T cells and high levels of IgG anti-chromatin, -ssDNA and -dsDNA antibodies [6], whereas B6.NZBc13 have impaired macrophage clearance of apoptotic debris and produce predominantly IgM and IgG anti-chromatin antibodies ([5], manuscript in preparation). In this study, we examined B6.NZBc1c13 bicongenic mice that contain both chromosomal intervals. Although these mice were originally produced to investigate the impact of genetic interactions between these loci on autoimmune phenotypes, the most striking finding in these mice was the marked expansion of their splenic myeloid (mDC) and plasmacytoid DC (pDC) populations. Despite these changes, only minor increases in ANA production were seen, and the severity of GN was reduced as compared to B6.NZBc1 mice.

As production of type I interferons (IFN) by IgG nuclear-antigen containing IC-stimulated plasmacytoid dendritic (pDC) has been proposed to play an important pathogenic role in SLE [7], we examined whether increased levels of IFN-α were seen in bicongenic mice. Surprisingly, splenic IFN-α levels remained low in bicongenic mice and production of IFN-α by pDC appeared to be reduced in older mice. This reduction in IFN-α production was not due to inhibition by other cytokines, such as TNF-α and IL-10, or regulation by other cellular populations. Instead, the pDC of older mice appeared to be refractory to TLR activation. Notably, both B6 and bicongenic bone marrow-derived pDC became refractory to TLR activation following repetitive stimulation with TLR ligands *in vitro*, demonstrating reduced secretion of IFN-α. This finding suggests that the lack of IFN-α production *in vivo* might arise from chronic activation due to nuclear antigens in the environment. Our results indicate that chronic exposure of pDC to IgG anti-nuclear IC does not necessarily lead to enhanced IFN-α production, and suggests that additional factors contribute to the abnormal production of IFN-α in human SLE.

## Results

### B6.NZBc1c13 mice demonstrate a dramatic expansion of DC populations

We have previously shown that B6.NZBc1 and/or B6.NZBc13 mice have a number of cellular abnormalities including splenomegaly, increased B and T cell activation, and expansion of mDC [4]–[5]. Therefore, the impact of genetic interactions between loci on chromosomes 1 and 13 on these phenotypes was assessed in bicongenic mice. As shown in Table 1, the splenic weight and number of splenocytes were significantly greater in 8-month-old bicongenic mice than their monocongenic counterparts, indicating that chromosomes 1 and 13 loci contribute additively to this phenotype.

**Table 1:** Comparison of the splenic phenotype in 8 month old B6.NZBc1c13 bicongenic mice with B6.NZBc1 and B6.NZBc13 congenic strains.

	B6	B6.NZBc1	B6.NZBc13	B6.NZBc1c13	NZB
	N = 18	N = 28	N = 9	N = 31	N = 9
Spleen weight (mg)	109.5±33.8	**222.4±56.7** ***	**246.4±130.9** **	**376.5±161.1**	**508.9±310.6**
# Splenocytes (×10^6^)	58.94±19.45	**100.3±48.32** *	**83.28±25.25** *	**131.8±65.49**	**136.5±84.07**
% B220^+^	56.48±9.02	58.16±8.28	59.78±7.61	51.65±10.01	**29.25±12.49** ***
% CD21^low^CD23^−^	6.97±2.33	8.14±3.05	**8.46±2.59**	7.69±3.26	**9.35±2.63**
% CD21^int^CD23^+^	38.61±10.09	40.49±9.15	35.36±8.03	34.15±11.90	**10.58±6.06** ***
% CD21^hi^CD23^+^	2.86±1.75	2.60±1.68	2.52±1.60	2.34±1.32	**0.30±0.38** ***
% CD21^hi^CD23^−^	3.67±1.22	**2.36±1.66**	**6.20±2.96** ***	**1.69±0.81**	**1.58±1.47**
% CD4^+^	19.68±3.96	20.61±2.76	18.77±3.25	20.70±3.75	23.16±5.00
% CD8^+^	10.91±5.43	**6.67±3.41**	8.96±1.13*	**5.77±2.18**	**6.07±2.61**
% CD11c^+^	10.33±4.47 (14)	14.36±6.53** (21)	12.91±4.69*	**23.22±9.81 (23)**	10.66±1.34***
% CD11b^+^CD11c^−^	2.38±1.06 (11)	3.52±1.45 (13)	1.54±0.73 (4)	2.42±1.08 (11)	**11.59±8.25** * **(8)**

Results are mean ± SD as determined by weight or flow cytometry. Significance level for comparison of B6.NZBc1c13 mice with other mouse strains was determined by Mann-Whitney non-parametric test,

* p<0.05,

** p<0.005,

*** p<0.0005. Numbers of 8 month old mice examined in each group are shown on the top unless otherwise indicated in brackets. Numbers shown in bold indicate significant difference p<0.05 from B6 control mice.

**Figure 1:**
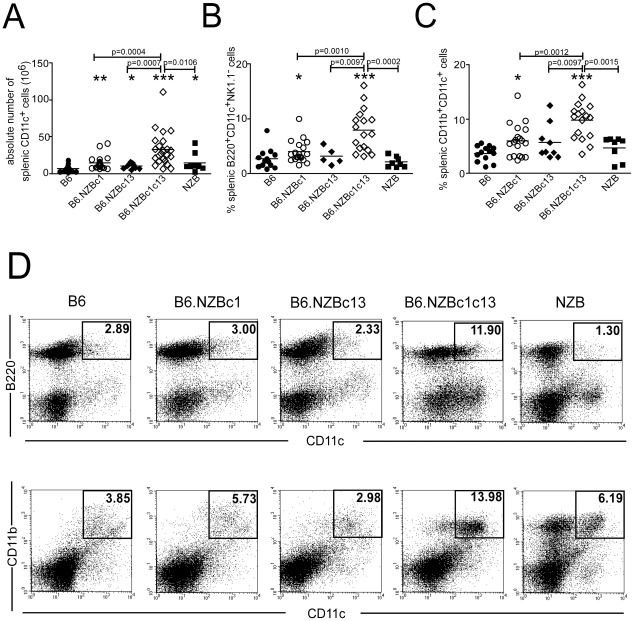
Expansion of dendritic cell populations in the bicongenic mice. Freshly isolated splenocytes from 8 month old female B6, B6.NZBc1, B6.NZBc13, B6.NZBc1c13 and NZB mice were stained with anti-CD11c in combination with anti-B220 and -NK1.1 or anti -CD11b antibodies to assess the proportion of plasmacytoid and myeloid DC. Shown are the (A) absolute number of splenic CD11c^+^ DC, and proportion of (B) B220^+^CD11c^+^NK1.1^−^ pDC and (C) CD11b^+^CD11c^+^ mDC. Each symbol represents the determination from an individual mouse. Horizontal lines indicate the mean for each population examined. The *p* values for significant differences between the congenic mouse strains are shown above bars, whereas asterisks represent significant differences between various congenic mice and B6 controls, *p<0.05, **p<0.005, ***p<0.0005. (D) Representative dot plots show the gating regions for NK1.1^−^ gated B220^+^CD11c^+^ pDC *(top panel)* and CD11b^+^CD11c^+^ mDC *(bottom panel)*. Numbers inside the box indicate the proportion of each population.

While the proportions of B220^+^, CD4^+^, CD8^+^ and CD11b^+^CD11c^−^ cells were similar in bicongenic mice to those observed in one or both monocongenic mouse strains, there was a marked increase in the proportion of CD11c^+^ cells (Table 1). In bicongenic mice, the proportion of CD11c^+^ cells was increased ∼2 fold as compared to both monocongenic strains and represented almost a quarter of splenocytes. To further characterize the phenotype of the expanded CD11c^+^ population(s), splenocytes were stained with anti-CD11c Ab together with anti-B220 and -NK1.1, or anti-CD11b to identify CD11c^+^B220^+^NK1.1^−^ pDC or CD11c^+^CD11b^+^ mDC, respectively. As shown in Figure 1, expansions of both pDC and mDC compartments contributed to the increased proportion of DC in bicongenic mice. This increase was most pronounced for pDC where there was a ∼5 fold increase in bicongenic mice as compared to monocongenic mice. As previously reported [8], increases in the splenic pDC and mDC compartment were not seen in NZB mice, suggesting that additional genetic loci present in NZB mice suppress this phenotype.

Expansion of the pDC population was not seen in the bone marrow of 8-month-old bicongenic mice (%CD11c^+^B220^+^NK1.1^−^ cells, B6 = 2.85%±1.34, n = 13; B6.NZBc1 = 2.72%±1.41, n = 18; B6.NZBc13 = 2.79%±1.51, n = 4; B6.NZBc1c13 = 2.29%±1.44, n = 17; NZB = 2.34%±1.09, n = 6; all p>0.05 as compared to B6 mice). However, moderate expansion of the bone marrow mDC compartment was observed (%CD11c^+^CD11b^+^ cells, B6 = 2.20%±1.24, n = 13; B6.NZBc1 = 4.01%±1.68, n = 18, p = 0.0035; B6.NZBc13 = 2.44%±1.35, n = 4, p>0.05; B6.NZBc1c13 = 5.57%±3.29, n = 17, p = 0.0002; NZB = 1.69%±0.59, n = 6, p>0.05, all p values as compared to B6). Differences in the proportions of pDC and mDC in the spleen, and for mDC in the bone marrow, were already seen in 2-month-old B6.NZBc1c13 mice but were much less marked (spleen pDC, B6 = 0.79%±0.33, n = 9; B6.NZBc1c13 = 1.34%±0.47, n = 11; p<0.05; spleen mDC, B6 = 2.53%±0.86, n = 9; B6.NZBc1c13 = 5.33%±1.49, n = 11; p<0.0005; bone marrow pDC, B6 = 2.09%±0.27, n = 6; B6.NZBc1c13 = 2.03%±0.49, n = 8; p>0.05 ; bone marrow mDC, B6 = 2.37%±0.29, n = 6; B6.NZBc1c13 = 3.36%±1.15, n = 8; p<0.05).

We have previously shown that 8-month-old B6.NZBc13 mice have B cell phenotypic changes similar to 4-month-old NZB mice, with reduced proportions of follicular (CD21^intermediate(int)^CD23^+^) and increased proportions of MZ (CD21^high(hi)^CD23^−^) and B1a (CD21^low(lo)^CD5^+^) B cells [5]. Surprisingly, in contrast to the DC changes, these phenotypes were not more pronounced in the bicongenic mice (Table S1). Indeed, B6.NZBc1c13 mice had reduced proportions of MZ B cells similar to B6.NZBc1 mice. This was not due to an age-associated loss of the MZ B cell population (as seen in NZB mice), because 4-month-old bicongenic mice demonstrated a similar reduction in their MZ B cell population (%CD21^hi^CD23^−^ cells, B6 = 5.89%±0.98, n = 15; B6.NZBc1 = 3.98%±1.84, n = 9, p = 0.0200; B6.NZBc13 = 9.76%±1.47, n = 2, p = N.D.; B6.NZBc1c13 = 4.38%±1.90, n = 11, p = 0.0430, all p values as compared to B6 mice). Nor was this reduction due to the presence of contaminating B220^+^ pDC, because the same changes were seen when this population was expressed as a proportion of total splenocytes (data not shown). Similar findings were observed for the splenic B1a cell population, where the proportion of cells in 8-month-old bicongenic mice was similar to that observed in B6.NZBc1 mice and was significantly reduced as compared to both B6.NZBc13 and NZB mice. Thus, the distribution of B cells in bicongenic mice appears to be driven predominantly by genetic loci on NZB chromosome 1, whereas genetic loci on chromosomes 1 and 13 interact additively to produce marked DC expansion in bicongenic mice.

Given the expansion of DC in bicongenic mice and the potential role of these populations in stimulation of B and T cells, we examined whether activation of these populations was increased in bicongenic mice. As shown in Table S1, activation of the CD21^int^, predominantly follicular, and CD21^hi^, precursor and mature MZ, B cell compartments was similar in bicongenic mice to that in parental monocongenic mouse strains. We were not able to examine activation in the CD21^lo^ B cell compartment, because this population was contaminated with pDC in our stains. The proportions of recently activated CD69^+^ and memory/effector CD44^hi^CD62L^lo^ CD4^+^ T cells in bicongenic mice were also not increased as compared to B6.NZBc1 mice. These findings indicate that the expansion of DC in bicongenic mice does not appear to lead to enhanced B or T cell activation.

### Clinical autoimmune disease is not amplified in bicongenic mice despite altered autoAb production

**Figure 2:**
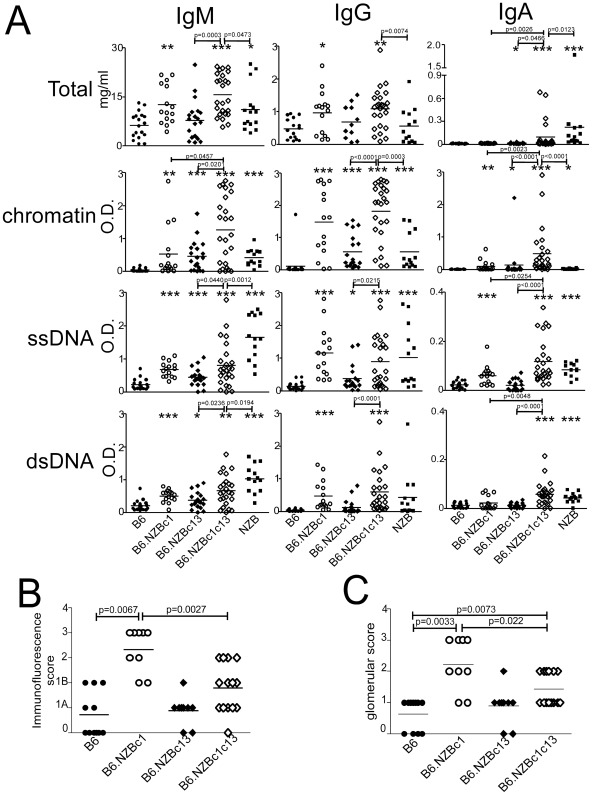
AutoAb levels and renal involvement in various congenic mouse strains. (A) Serum samples from 8 month old female B6, B6.NZBc1, B6.NZBc13, B6.NZBc1c13 and NZB mice were assayed for the presence of total IgM, IgG and IgA as well as IgM, IgG or IgA anti-chromatin, -ssDNA and -dsDNA Ab. (B) Immunofluorescence scores of frozen kidney sections stained with anti-IgG. Sections were graded as follows: grade 0, no or only trace deposits; grade 1, mesangial deposits (B more extensive than A); grade 2, mesangial and segmental capillary wall deposits; grade 3, diffuse mesangial and capillary wall deposits; grade 4, crescents. (C) Glomerular scores of kidneys fixed in formalin, paraffin embedded, sectioned, and stained with PAS. Sections were graded as: grade 0, normal glomeruli; grade 1, mesangial expansion and/or proliferation; grade 2, focal segmental proliferative glomerulonephritis; grade 3, diffuse proliferative glomerulonephritis; and grade 4, diffuse proliferative glomerulonephritis with crescents. Each symbol represents the determination from an individual mouse. Horizontal lines indicate the mean for each population examined. The *p* values for significant differences between the congenic mouse strains are shown above bars, whereas asterisks represent significant differences between the various congenic mice and B6 controls, *p<0.05, **p<0.005, ***p<0.0005.

To determine whether the expansion of DC in bicongenic mice is associated with more severe disease, autoAb levels and kidney disease were contrasted between the monocongenic and bicongenic mouse strains. Six- to 7-month-old B6.NZBc1 congenic mice produce significantly higher titers of IgG anti-histone, -chromatin and -ssDNA Ab than age-matched B6 controls, whereas IgM and IgG anti-chromatin Ab are predominantly produced in B6.NZBc13 mice [4]–[5]. As shown in Figure 2A, bicongenic mice demonstrated features of both strains, with elevated levels of IgG autoAb that approximated those seen in the B6.NZBc1 mouse strain and the increased levels of IgM anti-chromatin Ab seen in the B6.NZBc13 strain. In general, the levels of IgM autoAb in bicongenic mice exceeded those seen in the monocongenic mouse strains, which achieved statistical significance for anti-chromatin Ab. However, with the exception of anti-chromatin Ab, the levels of IgM autoAb remained lower than those seen in NZB mice. Notably, B6.NZBc1c13 mice produced moderate to high titers of IgA anti-chromatin Ab and low titers of IgA anti-ssDNA and -dsDNA Ab, which were markedly increased as compared to those seen in the monocongenic mice. Thus, genetic interactions between NZB chromosomes 1 and 13 result in the novel generation of IgA ANA.

Despite the development of these novel autoAb phenotypes and levels of IgG ANA similar to those observed in B6.NZBc1 mice, light microscopic changes and the amount of IgG deposition in the kidneys of bicongenic mice were significantly reduced compared to B6.NZBc1 and were similar to those observed for B6.NZBc13 mice (Figure 2B–C). Furthermore, although NZB chromosome 1 is reported to contain a genetic locus that facilitates anti-RBC Ab production in crosses with other lupus susceptibility loci [9], anti-RBC Abs were not produced in either B6.NZBc1 or B6.NZBc1c13 mice (%RBC IgM^+^, B6 = 1.39%±0.95 n = 4; B6.NZBc1 = 2.10%±0.87, n = 7; B6.NZBc1c13 = 2.23%±0.92, n = 8; all p>0.05 as compared to B6: %RBC IgG^+^, B6 = 0.67%±0.29, n = 4; B6.NZBc1 = 0.73%±0.46, n = 7; B6.NZBc1c13 = 0.77%±0.26, n = 8; all p>0.05 as compared to B6). Taken together, these findings indicate that the expansion of DC in bicongenic mice is not associated with a significant increase in the severity of the clinical disease phenotype.

### *In vivo* cytokine production in bicongenic mice

**Figure 3:**
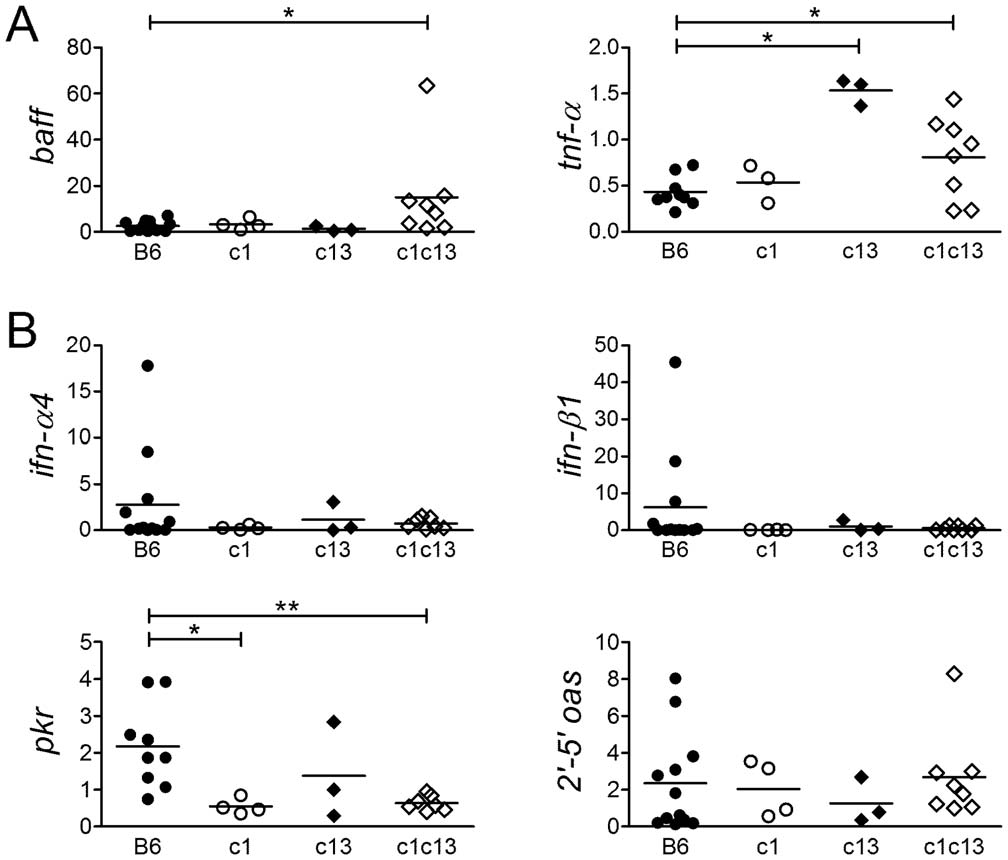
Production of excess BAFF and TNF-α, but reduced levels of IFN-α in the spleens of B6.NZBc1c13 mice. (A) Increased BAFF and TNF-α mRNA expression in B6.NZBc1c13 (c1c13) and B6.NZBc13 (c13) splenocytes and (B) reduced IFN- α/β and IFN-α-induced gene (*PKR* and *2′-5′ OAS*) expression in c1c13 and B6.NZBc1 (c1) splenocytes. Relative mRNA expression of genes of interest normalized to β-actin mRNA expression in freshly isolated splenocytes from 8-month-old female mice. Each point represents the determination from an individual mouse. The *p* values for significant differences between various congenic mice and B6 controls and various congenic mice are shown, *p<0.05, **p<0.005, as determined by Mann-Whitney non-parametric test.

One of the consequences of DC activation is the production of cytokines. Given the dichotomy between the expansion of the DC subsets and lack of exacerbated clinical autoimmunity in bicongenic mice, we examined the production of several pro-inflammatory cytokines, BAFF, TNF-α, and IFN-α, which are produced by DC and have been shown to play an important role in autoimmunity [10]–[12]. Since serum BAFF levels correlate poorly with BAFF production [13], splenic BAFF production was assessed by the measurement of *baff* mRNA levels using *q*RT-PCR and BAFF protein expression assessed using immunofluorescence microscopy. There was a ∼6 fold increase in *baff* mRNA levels in bicongenic as compared to B6 mice (Figure 3A, B6 = 2.58±2.24, n = 12; B6.NZBc1c13 = 14.93±20.31, n = 8), which was not seen in the parental congenic strains. Consistent with this observation, there were increased numbers of BAFF-producing cells in the spleens of bicongenic mice that were predominantly mDC (Figure S1). Similar to BAFF, there were increased levels of *tnf-α* mRNA in the spleens of bicongenic as compared to B6 mice (Figure 3A). However, the increase in *tnf-α* levels was only ∼2 fold (B6 = 0.43±0.17, n = 9; B6.NZBc1c13 = 0.81±0.45, n = 8). This appeared to arise from the B6.NZBc13 parental strain.

In contrast to BAFF and TNF-α, splenic levels of type I IFN and IFN-α-induced genes were normal or reduced in 8-month-old bicongenic as compared to B6 mice (Figure 3B). Similar findings were seen in the B6.NZBc1 parental strain. Thus, although pDC, the major producers of type I IFN, are increased in 8-month-old bicongenic mice, the levels of type I IFN are not increased. The lack of increased type I IFN and IFN-inducible gene expression in bicongenic mice was not due to insensitivity of the RT-PCR assay, because all of the values were well above the threshold of detection. A similar lack of increased expression of type I IFN or IFN-α-induced genes was also observed in the spleens of younger bicongenic mice (2-month-old) as well as the kidney and bone marrow of 9-month-old mice (Figure S2). However, a significant increase in *2′-5′ oas* was seen in the bone marrow of 2 month-old bicongenic mice.

### Reduced *in vitro* cytokine production by pDC from bicongenic mice

**Figure 4:**
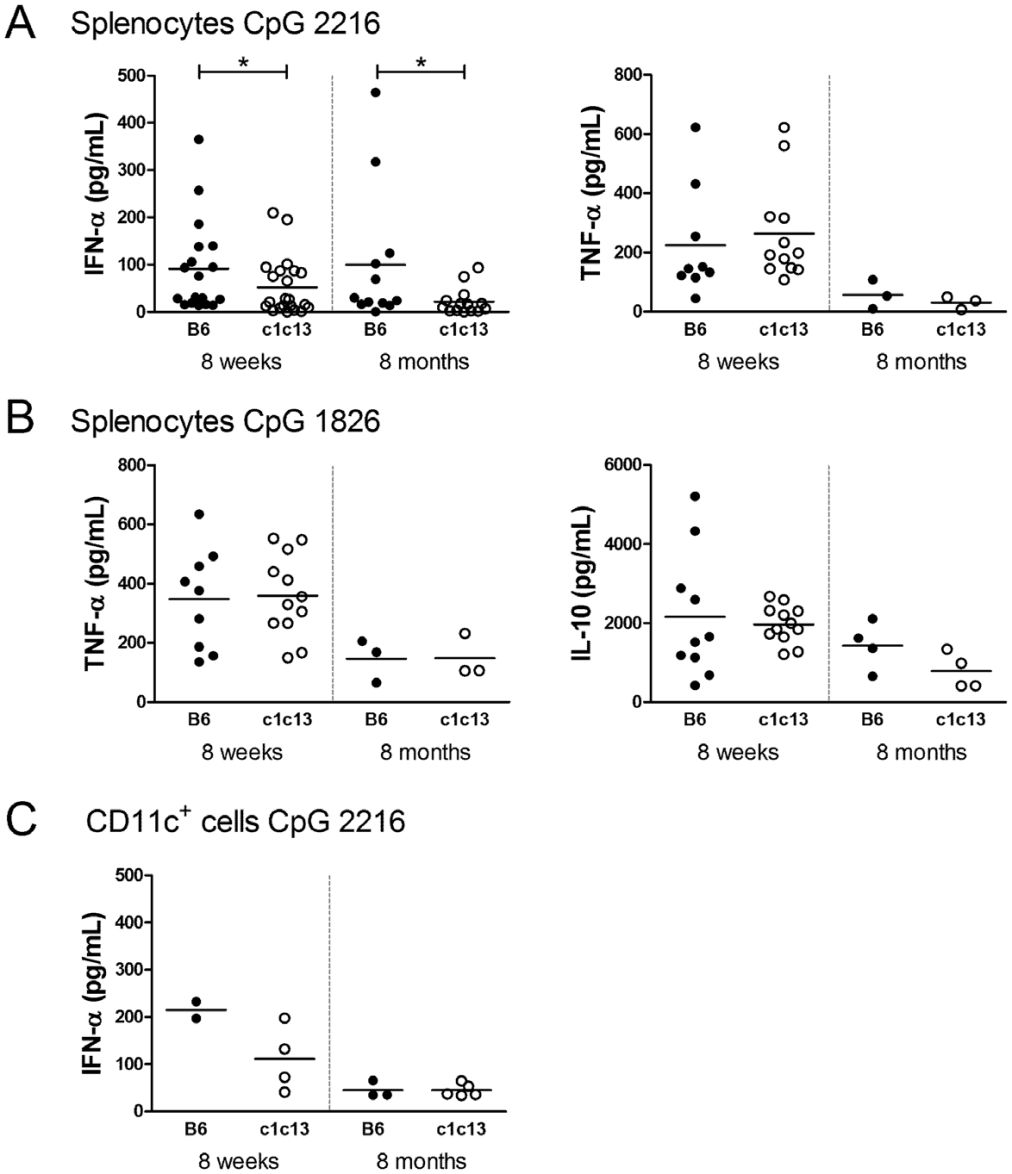
Reduced IFN-α, but not TNF-α and IL-10 production following stimulation of splenocytes with TLR ligands in bicongenic mice. Freshly isolated splenocytes from 8 week or 8 month old female B6 or B6.NZBc1c13 (c1c13) mice were stimulated with (A) CpG 2216 or (B) CpG 1826 for 48 h. Stimulation with CpG 2216 control or CpG 1826 control did not show differences between B6 and c1c13 mice (data not shown). (C) CD11c^+^ splenic DC from B6 and c1c13 mice were stimulated with CpG 2216 for 24 h. Levels of IFN-α, TNF-α and IL-10 in the culture supernatants were determined using ELISA. Each symbol represents the determination from an individual mouse. The *p* values for significant differences between B6 and B6.NZBc1c13 mice are shown above bars.

In lupus, pDC uptake of IgG IC containing DNA or RNA, results in TLR engagement and secretion of IFN-α [7], [14]–[16]. Thus, the relative absence of IFN-α secretion in bicongenic mice could reflect reduced activation of these cells as a consequence of low levels of anti-nuclear IC or an impaired ability of pDC to be stimulated by these complexes. Since bicongenic mice have increased levels of ANA and deposition of IC in their kidneys, it seemed unlikely that the absence of pDC IFN-α secretion in older mice resulted from a lack of IC; therefore, the ability of pDC to respond to TLR stimulation was assessed. To this end, freshly isolated splenocytes from 8-week- and 8-month-old mice were incubated with the TLR9 ligand, CpG 2216, which has been shown to induce IFN-α and TNF-α secretion by pDC [17], or CpG 1826, which has been shown to induce TNF-α and IL-10 secretion in a variety of cell types, as a control (Figure 4). Although there was considerable variability in the ability of splenocytes to secrete IFN-α following stimulation with CpG 2216 in both mouse strains, IFN-α production was generally reduced in bicongenic as compared to B6 mice. This was most marked in 8 month old mice, where reduced levels were seen despite a ∼5 fold expansion of the pDC population (Figure 4A). The variations in cytokine production between mice were not due to variations in the proportions of pDC, as reduced IFN-α secretion was seen in bicongenic mice with increased proportions of pDC, indicating reduced production on a per cell basis. Furthermore, reduced IFN-α secretion was still seen in some bicongenic mice when DC were isolated by positive selection (Figure 4C). Secretion of TNF-α by pDC was also reduced but this did not achieve statistical significance (Figure 4A). In contrast to the findings with CpG 2216, secretion of TNF-α and IL-10 secretion following CpG 1826 stimulation was preserved in 8 month-old bicongenic mice (Figure 4B).

To determine whether an intrinsic DC functional abnormality leads to the altered secretion of IFN-α by splenic DC stimulated with CpG 2216, bone marrow cells were obtained from 8–12-week-old B6 and pre-autoimmune bicongenic mice and cultured in the presence of Flt3L for 7 days to expand bone marrow-derived DC (BMDC). Although there were similar proportions of mDC and pDC in the bone marrow of young B6 and bicongenic mice pre-culture, and similar numbers of BMDC post-culture, there was a slight increase in the proportion of pDC and decrease in the proportion of mDC in BMDC cultures from bicongenic as compared to B6 mice (% pDC, B6 = 41.44%±1.20, n = 5; B6.NZBc1c13 = 46.33% ±2.64, n = 5, p = 0.0278; % mDC, B6 = 38.77%±2.31, n = 5; B6.NZBc1c13 = 31.10%±1.12, p = 0.0079). Immediately following expansion, basal levels of TLR (TLR3, TLR7, TLR9), MHCII, and B7.2 molecules were found to be similar for pDC and mDC derived from both strains (data not shown). Furthermore, BMDC from B6 and B6.NZBc1c13 mice produced similar amounts of IFN-α and IL-10 following stimulation with various TLR ligands, but demonstrated minor differences in the secretion of TNF-α with R837 and CpG 1826 (Fig. S3A–C). pDC from these strains of mice also demonstrated similar upregulation of B7.2 following stimulation with the TLR ligands (Fig. S3E). In contrast, B7.2 upregulation with the TLR3 ligand poly I:C and TLR9 ligands CpG 1826 and 2216 was reduced in mDC (Fig. S3F). Thus, pDC from BMDC appear to function normally, whereas some minor functional differences in mDC were observed between the two mouse strains. These findings suggest that the altered function of splenic pDC in bicongenic mice develops in response to the bicongenic environment.

### Reduced IFN-α secretion by pDC does not result from cytokine or cellular inhibition

**Figure 5:**
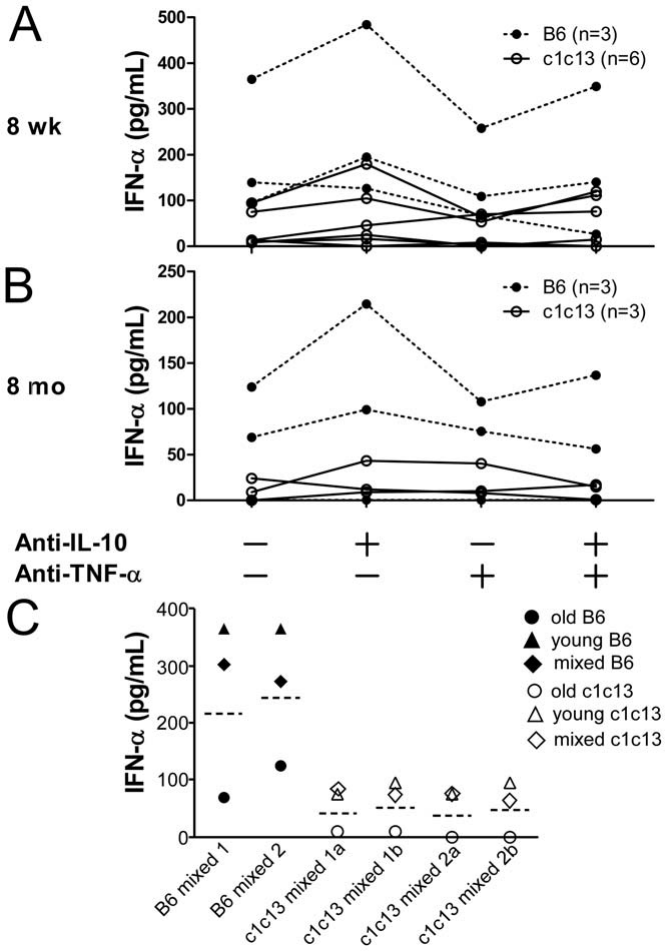
Cytokines or other cellular populations do not inhibit IFN-α production by CpG 2216 stimulated splenocytes from older c1c13 bicongenic mice. Freshly isolated splenocytes from (A) 8 week or (B) 8 month old female B6 or B6.NZBc1c13 (c1c13) mice were stimulated with CpG 2216 for 48 h in the presence or absence of anti-IL-10 and anti-TNF-α blocking antibodies. Levels of IFN-α in the culture supernatants were determined using ELISA. Each symbol represents the determination from an individual mouse. (C) Freshly isolated splenocytes from 8 to 12 week (young) and 8 month (old) B6 or c1c13 mice were stimulated alone or mixed at a 1∶1 ratio with CpG 2216 for 48 h. Levels of IFN-α production were determined by ELISA. The dotted lines represent the average IFN-α production by the young and old splenocytes when stimulated separately by CpG 2216. Shown in the figure are representative mice from two separate experiments.

Both IL-10 and TNF-α have been shown to inhibit IFN-α secretion by pDC [19]–[20], therefore it is possible that elevated levels of these cytokines in bicongenic mice inhibit IFN-α production. To investigate this possibility, IFN-α production was assessed in splenocyte cultures that were stimulated with CpG 2216 in the presence or absence of blocking antibodies against TNF-α and/or IL-10. As shown in Figure 5A and 5B, IL-10 blockade leads to a slight increase in IFN-α production in some mice, however this was similar in B6 and B6.NZBc1c13, and present in both young and old mice. In contrast, TNF-α blockade resulted in a slight decrease in IFN-α production in some mice. Blockade of both cytokines did not result in further augmentation of IFN-α production in either young or old mice. Thus, the reduction in IFN-α production by the splenocytes of older B6.NZBc1c13 mice does not appear to arise from inhibition by these cytokines.

As various cellular populations have also been shown to inhibit production of IFN-α by IgG IC-stimulated human pDC *in vitro* [21], we assessed whether splenocytes from older mice that demonstrated reduced production of IFN-α *in vitro* could inhibit production of IFN-α by splenocytes from young mice. To this end, splenocytes from young and old mice were mixed (1∶1) and IFN-α production was assessed following stimulation with ODN 2216 (Fig. 5C). In mixed cultures, cytokine production exceeded the average of those seen for splenocytes from young and old mice for both B6 and B6.NZBc1c13 strains, indicating that old cells were not inhibiting IFN-α production by young splenocytes. These findings suggest that other factors may be causing the reduced IFN production in old bicongenic mice.

### Bone marrow-derived pDC from bicongenic mice demonstrate normal cytokine production and tolerance following TLR stimulation in vitro

**Figure 6:**
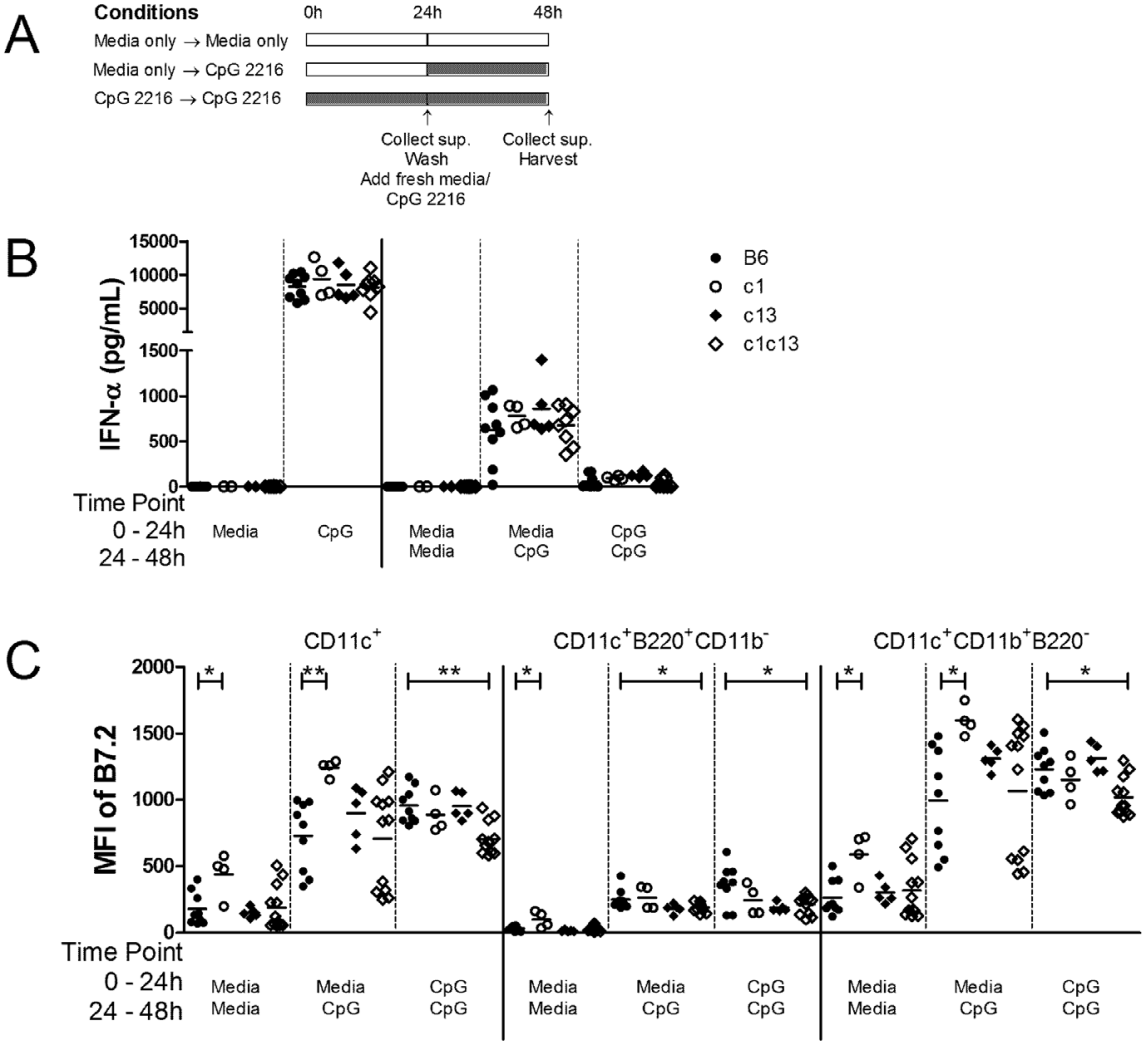
TLR tolerance impacts on IFN-α production, but not B7.2 upregulation, in BMDC from B6 and c1c13 bicongenic mice after repeated TLR9 stimulation. (A) Experimental design to investigate TLR tolerance of BMDC to TLR9. BMDC were cultured for 24 hours in the presence or absence of CpG 2216, washed and then stimulated again with CpG 2216. Supernatants were harvested at 24 (before washing) and 48 hours, and cellular profiles were assessed by flow cytometry after 48 hours. (B) IFN-α production by stimulated BMDC was measured by ELISA and (C) the mean fluorescence intensity (MFI) for B7.2 expression was quantified in various DC subsets (CD11c^+^ DC, CD11c^+^B220^+^CD11b^−^ pDC and CD11c^+^CD11b^+^B220^−^ mDC) using flow cytometry. Each symbol represents the determination from an individual female B6, B6.NZBc1 (c1), B6.NZBc13 (c13) or B6.NZBc1c13 (c1c13) mouse. Horizontal lines indicate the mean for each population examined. The *p* values for significant differences between various congenic mice and B6 control mice are shown, *p<0.05, **p<0.005.

Previous work on human pDC indicates that IFN-α secretion is inhibited by repetitive stimulation with TLR ligands or Ig IC-containing nuclear antigens, a phenomenon that has been termed TLR tolerance [22]–[23]. Thus, given the high titers of ANA together with the evidence of IC deposition in bicongenic mice, it is possible that the pDCs from older bicongenic mice are refractory to TLR stimulation as a consequence of chronic stimulation by IgG nuclear antigen-containing IC. To explore this possibility, we examined activation of BMDC following single and repetitive stimulation with CpG 2216. To this end, BMDC were cultured for 24 hours in the presence or absence of CpG 2216, washed and then stimulated again with CpG 2216. Supernatants were harvested at 24 (before washing) and 48 hours, with cell viability and cellular activation (upregulation of B7.2) being measured at the endpoint by flow cytometry (Fig. 6A). Although pDC stimulated after 24 hours secreted significantly lower levels of IFN-α than those stimulated at the onset of culture period, the levels of IFN-α secreted by pDC from all mouse strains tested were comparable (Fig. 6B). Notably, IFN-α secretion in the second 24-hour period of culture was completely abrogated by incubation with CpG 2216 in the first 24 hours, with similar findings for B6 and B6.NZBc1c13 pDC. In contrast, B7.2 expression was maintained or further increased by a second stimulation with CpG 2216, suggesting that the cells are not refractory to activation but that cytokine secretion may be specifically inhibited (Fig. 6C). The reduced cytokine production was not secondary to reduced cell viability because the proportion of live cells was similar in both cultures. Thus, murine pDC like their human counterparts, demonstrated impaired IFN-α production following repeated TLR stimulation and this process is intact in bicongenic mice.

### Splenic pDC in older bicongenic mice have a phenotype suggesting chronic activation *in vivo*

**Figure 7:**
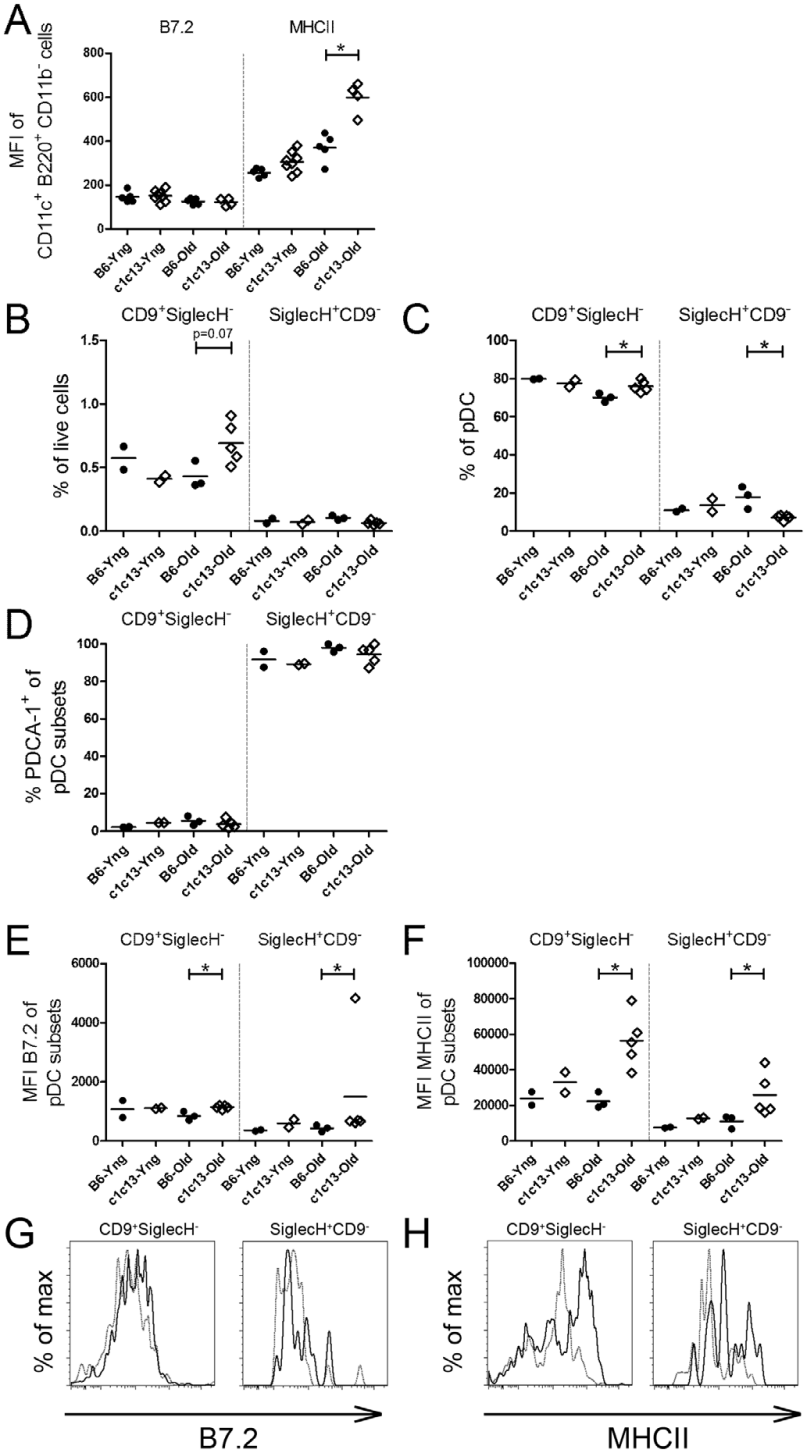
Increased activation of cells in the immature and mature pDC subsets of older bicongenic mice. Expression of the activation markers (A) B7.2 and MHCII was quantified by mean fluorescent intensity (MFI) on splenic pDC gated as CD11c+ B220+ CD11b− in 8 week old (young; Yng) and 8 month old (Old) B6 and bicongenic mice. pDC populations were further characterized with additional surface markers to remove contaminating IgM+ CD19+ ABCs and to determine the maturity of pDC. Immature (CD9+ SiglecH−) and mature (SiglecH+ CD9−) pDC were gated on the CD11c+ B220+ CD11b− IgM− CD19− splenic pDC population and the data expressed as a proportion of total live splenocytes (B) and as a proportion of total pDC (C). (D) PDCA-1 was highly expressed on mature (SiglecH+ CD9−) but not immature (CD9+ SiglecH−) pDC subsets. Elevated expression of activation markers (E) B7.2 and (F) MHCII was seen in older bicongenic mice in both pDC subsets gated from the CD11c+ B220+ cell population. Representative histogram plots for older B6 (grey dotted line) and bicongenic mice (solid black line) for (G) B7.2 and (H) MHCII were gated on the CD11c+ B220+ CD11b− cells population excluding ABCs, in both the CD9+SiglecH− and SiglecH+CD9− populations. Results from this figure show representative data from one of three independent experiments. The p values for significant differences between B6 and bicongenic mice are shown, *p<0.05.

The findings outlined above raised the possibility that chronic stimulation of pDC by nuclear antigen-containing IC in bicongenic mice leads to their failure to secrete IFN-α in bicongenic mice. To address this possibility, expression of B7.2 and MHC Class II (MHC II), two molecules that are increased with activation, was examined in freshly isolated splenocytes from young and old mice. Since the CD11c^+^ B220^+^ NK1.1^−^ population that was used to gate pDC could be contaminated with age-associated B cells (ABCs), which have been shown to express similar markers and express high levels of B7.2 and MHC II, additional stains were performed to determine the proportion of these cells within the gated population and to enable examination of activation of pDC in the absence of the contaminating ABCs [18]. By definition the ABC subset is CD11c^int^CD11b^int^ and expresses CD19 and IgM. Approximately 10–20% of the cells gated within the conventional pDC gate expressed these markers, which was similar for both B6 and bicongenic, young and old mice (Fig. S4A). An increased proportion of pDC (CD11c^+^ B220^+^ CD11b^−^) was still seen in old bicongenic mice when CD11b^+^ cells, including the ABC subset, were excluded from the analysis (Fig. S4B). As shown in Figure 7A, increased levels of MHC II, but not B7.2, were seen on the pDC of older bicongenic mice, using this more restrictive gating.

Interestingly, the ABC subset was also elevated in older bicongenic mice (Fig. S4C–D).

Previous studies have also shown that there are distinct subpopulations within the pDC subset that vary in their capacity to secrete IFN-α. Thus, the impaired ability of splenic pDC from bicongenic mice to secrete IFN-α following stimulation might reflect over-representation of pDC populations that cannot secrete IFN-α. To examine this possibility, freshly isolated splenocytes from young and old mice were stained with anti-CD11c, B220, CD11b, IgM, and CD19, to permit gating of pDC free of ABCs, in tandem with anti-CD9 and SiglecH. It has been shown that the less mature CD9^+^ SiglecH^−^ pDC can secrete IFN-α, whereas the more mature SiglecH^+^ CD9^−^ pDC cannot [24]. As shown in Figure 7B and 7C, older bicongenic mice have an increased number and proportion of immature pDC, a phenotype suggesting they should have the capacity to secrete IFN-α. The PDCA-1 staining in these each of these populations, was similar to that previously reported, and did not differ between the mouse strains (Figure 7D). Notably, both the CD9^+^ SiglecH^−^ and SiglecH^+^ CD9^−^ subpopulations of pDC showed evidence of increased activation in older bicongenic mice (Figure 7E & 7F). As shown in Figure 7G & 7H, increased expression of B7.2 and MHC II was seen also in the CD11b^−^ subset of these subpopulations. Thus, the reduced levels of IFN-α in bicongenic mice appear to result from an impaired ability of previously stimulated immature pDC to secrete IFN-α.

## Discussion

In this paper, bicongenic mice with NZB chromosome 1 and 13 intervals were produced to determine whether genetic interactions between these two chromosomes lead to exacerbated autoimmunity. While autoimmunity was not more pronounced in these mice than in parental monocongenic strains, several novel phenotypes were seen that were either absent in the monocongenic strains or markedly exacerbated in bicongenic mice. These included dramatic increases in the size of the mDC and pDC populations, and elevated levels of total and anti-nuclear IgA antibodies.

Although there are small but significant increases in the proportions of mDC and pDC for B6.NZBc1 and mDC in B6.NZBc13 mice, these were markedly augmented in the bicongenic mice. As yet, the precise mechanisms leading to this increased expansion in bicongenic mice have not been determined. However, similar expansions of these subsets have been seen in mice with increased TLR responses and/or impaired clearance of apoptotic debris such as TLR7 transgenic [25], Tir8 knockout [26], and Mer^Kd^ [27] mouse strains. Increased TLR signaling can produce expansion of DC populations either by directly inducing activation or migration of DC into the spleen [28]–[29], or indirectly by increasing pro-inflammatory factors that have been shown to recruit and/or promote survival of DC [30]–[32]. It is likely that the expansion of DC in bicongenic mice results from a combination of these factors. Recent experiments in our laboratory indicate that B6.NZBc13 mice have a defect in clearance of apoptotic debris by macrophages, resulting in increased availability of TLR-stimulating nuclear antigens (manuscript in preparation). Although splenic pDC from mice with a shorter NZB chromosome 1 interval encompassed within the interval studied here have been reported to secrete augmented levels of IFN-α and IL-10 in response to TLR-9 stimulation suggesting an increased TLR response [33], this phenotype was not seen in bicongenic mice. However, bicongenic mice have increased amounts of a variety of pro-inflammatory factors including IFN-γ, TNF-α and BAFF, raising the possibility that the expansion of DC in these mice may arise from increased amounts of pro-inflammatory factors rather than TLR-hyper-responsiveness. It is possible that this capacity to produce pro-inflammatory factors arises from genetic loci on chromosome 1, as increased levels of these cytokines, with the possible exception of TNF-α, were not seen in B6.NZBc13 mice. Thus, the expansion of pDC and mDC in bicongenic mice may arise from increased amounts of apoptotic debris arising from a genetic locus on NZB chromosome 13, acting in tandem with increased production of pro-inflammatory factors arising from genetic loci on NZB chromosome 1.

BAFF levels were markedly increased in bicongenic mice as compared to B6 mice and were at least as high as those seen in NZB mice. This increase appeared to require genetic loci from both chromosomes 1 and 13, as it is seen in neither B6.NZBc1 nor B6.NZBc13 8-month-old mice. In BAFF transgenic mice, there are increased levels of total serum IgA and IgA autoAb, and BAFF has been shown to facilitate class switching to IgA production [34]–[35]. Thus, it is likely that the increased IgA levels in NZB and bicongenic mice arise, at least in part, from increased BAFF. However, the elevated levels of BAFF in bicongenic mice were not sufficient to overcome the reduction in the MZ B cell population mediated by genetic loci on chromosome 1. At present, it is unclear what is driving this BAFF production in bicongenic mice. Although IFN-α has been shown to enhance BAFF production [36], the low levels of IFN-α and IFN-induced gene expression in bicongenic mice suggest that IFN-α does not play a significant role in BAFF induction. Uptake of circulating Ag, such as apoptotic debris, has also been shown to promote localization of mDC-like cells to the spleen [37] and induce their BAFF expression [16], [37]. These cells have been shown to localize initially to the MZ, where they cluster with B cells, inducing their Ab secretion. In bicongenic mice, BAFF-producing cells are scattered throughout the red pulp and are not found in clusters with Ab-producing B cells (Figure S1), however it is possible that their localization in the MZ was a transient state or that they were already localized in the spleen and became activated *in situ*.

Despite the marked expansion of the pDC subset in bicongenic mice, levels of type I IFN did not appear to be increased. This may be relevant to the lack of severe GN in the bicongenic mice, since even modest increases in IFN-α have been shown to markedly accelerate kidney disease [10]. Several possible mechanisms were explored for the lack of IFN-α in older bicongenic mice. Both cytokines and direct cellular interactions have been shown to modulate IFN-α secretion by pDC. However, blocking studies with monoclonal antibodies directed against TNF-α and IL-10, two cytokines shown to inhibit IFN-α secretion by pDC [19]–[20], did not restore the capacity of pDC from older mice to secrete IFN-α. In mixing experiments, splenic cellular populations from older mice also had no impact on IFN-α production by pDC from young mice. Thus, the secretion of IFN-α in older bicongenic mice does not appear to be actively suppressed by either cytokines or cellular interactions. Chronic activation of human pDC by TLR ligands has been shown to lead to desensitization to TLR-signaling, resulting in impaired production of IFN-α. To determine whether murine pDC can be rendered similarly refractory to activation, BMDC were stimulated repeatedly with a TLR9 ligand. Similar to their human counterparts, repeated stimulation of pDC led to markedly impaired IFN-α secretion. This raised the possibility that chronic stimulation of pDC by nuclear antigen-containing IC in bicongenic mice leads to their failure to secrete IFN-α in bicongenic mice. Consistent with this possibility, pDC of older bicongenic mice showed evidence of previous activation *in vivo*, with increased expression of MHCII, as compared to B6 mice. This increased activation was seen both in the immature and mature pDC populations. In contrast to MHCII, the levels of B7.2 were either not or only marginally increased on pDC in older bicongenic mice. As shown in Figure 6, pDC from bicongenic mice are impaired in their ability to upregulate B7.2 following TLR stimulation as compared to those from B6 mice. Thus the relative lack of increased B7.2 expression in older bicongenic mice could reflect this signaling difference. Notably, there was an increased proportion and number of immature pDC in older bicongenic mice. This finding suggests that the impaired ability of pDC to secrete IFN-α in older bicongenic mice does not result from a decreased proportion of immature pDC, but instead appears to reflect the prior activation of these cells *in vivo*. In further support of the concept, the only site at which there is evidence of an IFN-α signature in bicongenic or NZB mice [38] is in the bone marrow, the organ at which pDC are first exposed to apoptotic debris.

Regardless of the mechanisms leading to impaired IFN-α secretion in bicongenic mice, the data reported herein indicate that expansion of pDC and the presence of anti-DNA Ab need not be associated with increases in IFN-α production. In humans with SLE, IgG anti-DNA immune complexes have been proposed to drive the abnormal production of IFN-α by pDC. However, our findings suggest that there must be additional complexity in the generation and regulation of IFN-α beyond this mechanism. Further characterization of these processes may provide important insights into the immune dysregulation that promotes SLE.

## Materials and Methods

### Ethics statement

Mice were housed in a Canadian Council on Animal Care approved facility at the Toronto Western Research Institute, part of the University Health Network. All mice used and experiments performed in this study were approved by the Animal Care Committee of the University Health Network (Animal Use Protocol #123).

### Mice

B6 and NZB mice were purchased from Taconic Farms (Germantown, NY) and Harlan-Sprague-Dawley (Blackthorne, England), respectively, and subsequently bred in our facility. Congenic mice were produced by separately backcrossing NZB chromosome 1 and 13 intervals onto the B6 background, using the speed congenic technique [39]. Mice were genotyped at each successive generation using polymorphic microsatellite markers that discriminate between NZB and B6 DNA, spaced at ∼20 cM intervals throughout the genome, except for regions containing lupus susceptibility genes where more densely spaced markers were used. Fully backcrossed mice were produced within 6 or 7 generations, for chromosome 1 and 13 intervals respectively, and then intercrossed to produce congenic mice that were homozygous for the NZB intervals. For NZB chromosome 1 congenic mice (previously called B6.NZBc1(35-106) but here denoted as B6.NZBc1 for simplicity) the NZB interval extends from between rs13475886 (61.2 Mb) and D1Mit303 (63.0 Mb) to between D1Mit223 (190.5 Mb) and D1Mit210 (192.1 Mb). NZB chromosome 13 congenic mice (B6.NZBc13) have an NZB interval extending from between D13Mit117 (37.7 Mb) and D13Mit92 (46.9 Mb) to D13Mit78 (119.6 Mb). B6.NZBc1c13 bicongenic mice were produced by intercrossing B6.NZBc1 and B6.NZBc13 mice and selecting for homozygous NZB chromosome 1 and 13 intervals in successive crosses. Typing of all polymorphic markers (spaced on average ∼5–6 Mb apart) in the NZB chromosome 1 and 13 intervals of bicongenic mice was identical to that of the parental monocongenic mice. The mice were housed in microisolators in the animal facility at the Toronto Western Hospital (Toronto, Canada) and were specific-pathogen free. All of the mice that were examined in this study were female.

### Flow cytometry analysis

RBC-depleted splenocytes, bone marrow cells, or BMDC (5×10^5^) were incubated with 10 µg/ml mouse IgG (Sigma-Aldrich, St Louis, MO) for 15 min to block Fc receptors and stained with various combinations of directly-conjugated mAbs. Following washing, allophycocyanin-conjugated streptavidin (BD Biosciences, San Diego, CA) was used to reveal biotin-conjugated Ab staining. Dead cells were excluded by staining with 0.6 µg/ml propidium iodide (PI; Sigma-Aldrich). For intracellular staining, cells were fixed and permeabilized using Cytofix/Cytoperm (BD Biosciences), and washed and stained in Perm/Wash buffer (BD Biosciences), according to the manufacturer's protocol. Flow cytometry of the stained cells was performed using a FACSCalibur or LSRII (BD Biosciences, Mountain View, CA) and analyzed using Cell Quest Pro (BD Biosciences) or FlowJo (Treestar) software. Live cells were gated on the basis of PI exclusion and scattering characteristics, with 10,000 or 25,000 events being acquired for each sample. The following directly conjugated mAbs were purchased from BD Biosciences: biotin conjugated anti-CD11c (N418), -CD11b (Mac1), -CD4 (L3T4), -CD8 (53-6.7), and -CD62L (MEL-14); PerCP conjugated anti-B220 (30-F11); PE conjugated anti-B7.1 (16-10A1), -B7.2 (GL1), -IA/IE (M5/114.15.2), -CD3 (145-2C11), -CD23 (B3B4), -IgM^b^ (AF6-78), -CD69 (H1.2F3), -CD44 (IM7), -ICAM-1 (3E2), -NK1.1 (PK136), and -CD4 (H129.19); FITC conjugated anti-CD3 (145-2C11), -CD4 (L3T4), -CD8 (53-6.7), -CD21/CD35 (7G6), -CD25 (7D4), -B220 (RA3-6B2), and -CD11c (N418); PE-Cy7 conjugated anti-CD19 (1D3); Pacific Blue conjugated anti-B220 (RA3-6B2); and APC-Cy7 conjugated anti-CD11b (M1/70). Allophycocyanin conjugated anti-CD9 (eBioKMC8) was purchased from eBioscience, and Pacific Blue anti-SiglecH (551) and APC-Cy7 anti-IA/IE (M5/114.15.2) were purchased from BioLegend. Biotinylated antibiodies were revealed with streptavidin conjugated with allophycocyanin or PerCP from BD Biosciences. FITC and biotin conjugated PDCA-1 (Miltenyi Biotec) was a generous gift from Dr. Eleanor Fish. FITC anti-CD62L (MEL-14) and anti-CD11b (M1/70.15) were purchased from Cedarlane (Hornby, Ontario, Canada). All isotype controls, with the exception of hamster IgG controls (BD Biosciences), were purchased from Cedarlane.

### Measurement of Ab production

Serum levels of IgM, IgG, and IgA anti-chromatin, -dsDNA and -ssDNA Ab were measured by ELISA. ssDNA was prepared by boiling dsDNA (isolated from calf thymus DNA) for 10 min and quick cooling on ice for 2 minutes. H1-stripped chromatin was prepared from chicken RBC, as previously described [40]. ELISA plates were coated overnight with chromatin (8 µg/ml), dsDNA (40 µg/ml) or ssDNA (20 µg/ml) diluted in PBS at 4°C. The plates were then washed with 0.05% Tween 20/PBS, and blocked with 2% BSA/PBS for 1 h at room temperature. After further washing, serum samples, diluted 1/100 in 2% BSA/Tween 20/PBS, were added. Bound Ab were detected using alkaline phosphatase-conjugated anti-IgG, -IgM or -IgA Ab (Caltag, Burlingame, CA) as secondary reagents. For measurement of total IgM, IgG, and IgA, plates were coated with goat anti-mouse IgM or IgG (Jackson ImmunoResearch, West Grove, PA, USA) or rat anti-mouse IgA (BD Biosciences) respectively, and the serum was diluted 1/1000. The amount of bound IgM, IgG or IgA was calculated from a standard curve using purified class-specific controls, and the Ab concentration was calculated from a plot of concentration versus absorbance.

### Bone marrow-derived DC (BMDC) cultures and CD11c^+^ splenic DC isolation

Bone marrow cells were isolated by flushing femurs of 8–12 week-old mice. After RBC lysis, *ex vivo* cells were resuspended to 10^6^ cells/mL and cultured for 7 days with recombinant human Flt3L (20 ng/mL; R&D Systems) in complete RPMI 1640 media (10% fetal bovine serum (FBS), non-essential amino acids, L-glutamine, β-mercaptoethanol, penicillin, and streptomycin). Splenic DC were positively selected using biotin-conjugated anti-CD11c antibody (N418) and Dynabeads Biotin Binder kit (Invitrogen), following the manufacturer's instructions.

### TLR stimulation and cytokine blockade

TLR ligands used were imiquimod R837 (2 µM; TLR7), poly I:C (50 µg/mL; TLR3), CpG ODN 2216 or control (250 nM; TLR9), CpG ODN 1826 or control (250 nM; TLR9) all from InvivoGen (San Diego, CA), and LPS (25 µg/mL; Sigma-Alderich) as a positive control. For splenocytes cultures, 2×10^6^ cells were cultured in 96-well flat-bottom plates for 48 hours with various TLR ligands or in media alone (0.5% normal mouse serum in complete RPMI1640 without FBS). For BMDC cultures, 4×10^5^ cells were cultured in 96-well flat-bottom plates for 24 or 48 hours with various TLR ligands or in media alone (10% FBS in complete RPMI 1640). For CD11c^+^ splenic DC cultures, 2×10^5^ cells were cultured in 96-well flat-bottom plates for 24 hours with CpG 2216 or in media alone (10% FBS in complete RPMI 1640). To assess IFN-α, IL-10 and TNF-α production following TLR stimulation, cytokine levels in tissue culture supernatants were measured by ELISA using commercially available kits as follows: IFN-α, PBL Biomedical Laboratories (Piscataway, NJ); IL-10, SABiosciences Corporation (Frederick, MD) or eBioscience (San Diego, CA); and TNF-α, BD Biosciences or eBioscience. Assays were performed as per the manufacturer's recommendations with the concentration of cytokine calculated from a standard curve of absorbance versus concentration of recombinant cytokine.

### Immunofluorescence staining of tissue sections

Spleens were snap frozen in OCT compound (Sakura Finetek, Torrance, CA) at the time of sacrifice. Cryostat sections (5 µm) were fixed in acetone, washed with PBS, and blocked with 5% normal goat serum/PBS or 5% fetal bovine serum/PBS. Sections were stained with biotinylated anti-CD11b and FITC anti-CD11c to detect myeloid DC. BAFF production was assessed by staining in tandem with rabbit IgG anti-BAFF (Sigma) followed by AMCA-conjugated goat anti-rabbit IgG Ab (Jackson ImmunoResearch). Biotin staining was revealed using rhodamine-conjugated streptavidin as a secondary reagent (Molecular Probes, Eugene, OR). Stained sections were mounted with Mowiol (Calbiochem, La Jolla, CA) and tissue fluorescence visualized using a Zeiss Axioplan 2 imaging microscope (Oberkochen, Germany). Digital images were obtained using the manufacturer's imaging system.

### Grading of kidney sections

For immunofluorescence score, kidneys were snap frozen in OCT compound (Sakura Finetek) and sectioned (3 µm). For glomerular score, kidneys were fixed in formalin, paraffin embedded, sectioned (3 µm), and stained with periodic acid-Schiff. Grading was performed by a renal pathologist (G. Lajoie) who was blinded as to the strain of origin of the tissue section. Glomerular staining of kidney sections stained with FITC anti-IgG was graded by immunofluorescence microscopy. Sections with no or only trace deposits were graded as 0; those with mesangial deposits, grade 1 (B more extensive than A); those with mesangial and segmental capillary wall deposits, grade 2; those with diffuse mesangial and capillary wall deposits, grade 3; and those with crescents, grade 4. The grading scale used for glomerular score using light microscopy was as follows: grade 0, normal glomeruli; grade 1, mesangial expansion and/or proliferation; grade 2, focal segmental (endocapillary) proliferative glomerulonephritis; grade 3, diffuse (endocapillary) proliferative glomerulonephritis; and grade 4, diffuse proliferative glomerulonephritis with crescents.

### Measurement of mRNA expression

RNA was purified from splenocytes and bone marrow cells of 8-month-old mice using the RNeasy Mini Kit (Qiagen, Basel, Switzerland), treated with DNaseI (Invitrogen, Canada), and converted to cDNA using the High Capacity cDNA Reverse Transcription Kit (Applied Biosystems, Forster City, CA), according to the manufacturer's instructions. Quantitative real-time PCR was performed with SYBR Green Master Mix on a 7900HT Fast Real-Time PCR System (Applied Biosystems) using default cycling conditions. Primer sequences were designed to span exon-to-exon and were as follows: *β-actin* forward, TTGCTGACAGGATGCAGAAG, *β-actin* reverse, GTACTTGCGCTCAGGAGGAG; *baff* forward, CAGGAACAGACGCGCTTTC, *baff* reverse, GTTGAGAATGGCGGCATCC; *tnf-α* forward, GCCACCACGCTCTTCTGTCT, *tnf-α* reverse, TCTGGGCCATAGAACTGATGAGA; *pkr* forward, TGAGCGCCCCCCATCT, *pkr* reverse, TATGCCAAAAGCCAGAGTCCTT; *2′-5′ oas* forward, TGAGCGCCCCCCATCT, *2′-5′ oas* reverse, CATGACCCAGGACATCAAAGG; *ifn-α4* forward, CTTGTCTGCTACTTGGAATGCAA, *ifn-α4* reverse, AGGAGGTTCCTGCATCACACA; and *ifn-β1* forward, TGACGGAGAAGATGCAGAAGAG, *ifn-β1* reverse CACCCAGTGCTGGAGAAATTG. Gene expression was analyzed using the relative standard curve method and was normalized to *β-actin* expression.

### Statistics

Statistical significance of comparisons between groups of mice was determined using the Mann-Whitney non-parametric two-tailed test with the exception of comparisons between kidney tissue section grades where Fisher's exact test was used.

## Supporting Information

Table S1 **Comparison of the B and T cell phenotypes in 8 month old B6.NZBc1c13 bicongenic mice with B6.NZBc1 and B6.NZBc13 congenic strains.** Results are mean ± SD as determined by flow cytometry. Significance level for comparison of B6.NZBc1c13 mice with other mouse strains was determined by Mann-Whitney non-parametric test, *p<0.05, **p<0.005, ***p<0.0005. Numbers of 8 month old mice examined in each group are shown on the top unless otherwise indicated in brackets. Numbers shown in bold indicate significant difference p<0.05 from B6 control mice.
(DOC)

Figure S1 **Splenic BAFF expression in B6 and c1c13 bicongenic mice.** Cell populations producing BAFF were characterized by staining with biotinylated anti-CD11b, and FITC anti-CD11c with rabbit IgG anti-BAFF followed by AMCA-conjugated goat anti-rabbit IgG Ab. Biotin staining was revealed using rhodamine-conjugated streptavidin as a secondary reagent. Arrows indicate the same BAFF-producing CD11b^+^CD11c^+^ mDC in each image. The BAFF-producing CD11c^+^ cells did not stain with B220 (data not shown). Scale bar, 100 µm.
(TIF)

Figure S2 **IFN-α/β and IFN-α-induced gene expression in various organs of 2 and 9 month-old B6 and bicongenic mice.** Expression of IFN-α/β and IFN-α-induced (*PKR* and *2′-5′ OAS*) genes was measured by *q*RT-PCR in B6 and B6.NZBc1c13 (c1c13) spleen (A) and bone marrow cells (B) at 2 months of age, and in the bone marrow cells (C) and kidneys (D) at 9 months of age. Relative mRNA expression of genes of interest normalized to β-actin mRNA expression. Each point represents the determination from an individual mouse. The *p* values for significant differences between B6.NZBc1c13 and B6 controls by Mann-Whitney non-parametric test were shown, *p<0.05.
(TIF)

Figure S3 **Similar levels of cytokine production, but reduced B7.2 expression in myeloid dendritic cells from bicongenic mice upon TLR stimulation.** BMDC from 8–12 week-old mice were expanded in the presence of Flt3L for 7days and then cultured in the presence or absence of imiquimod R837, poly I:C, CpG 1826, CpG 2216 and LPS. (A) IFN-α, (B) TNF-α, and (C) IL-10 production in the culture supernatant was measured by ELISA. Each symbol represents the determination from an individual mouse, with background levels of cytokine with media alone subtracted. MFI for B7.2 expression on (D) CD11c^+^ total dendritic cells, (E) CD11c^+^B220^+^CD11b^−^ plasmacytoid dendritic cells, and (F) CD11c^+^CD11b^+^B220^−^ myeloid dendritic cells, as determined by flow cytometry. The p values for significant differences are shown, where *p<0.05, **p<0.005, ***p<0.0005, and were determined by the Mann-Whitney non-parametric test. Horizontal lines indicate the mean for each population examined.
(TIF)

Figure S4 **Examination of the splenic age-associated B cell population.** (A) Similar proportion of contaminating IgM^+^ CD19^+^ CD11b^int^ cells within the CD11c^+^ B220^+^ cell population across all strains and age groups. (B) Elevated levels of pDC in older bicongenic mice gated as CD11c^+^ B220^+^ CD11b^−^ and excluding CD11b^int^ ABC subset. (C) Increased proportion of ABCs is seen only in older bicongenic mice. ABCs were gated as IgM^+^ CD19^+^ B220^+^ CD11c^int^ CD11b^int^ and are expressed as a percentage of live cells. (D) Representative contour plots showing the gating and proportion of CD11c^int^ CD11b^int^ cells as a percentage of live cells in young and old B6 and bicongenic mice. The p values for significant differences are shown, where *p<0.05, ***p<0.0005, and were determined by the Mann-Whitney non-parametric test.
(TIF)
