# Supplementary material for: TLR Tolerance Reduces IFN-Alpha Production Despite Plasmacytoid Dendritic Cell Expansion and Anti-Nuclear Antibodies in NZB Bicongenic Mice
Source: PLoS One. 2012 May 4;7(5):e36761. doi: 10.1371/journal.pone.0036761 (PMC3344944; doi:10.1371/journal.pone.0036761)
Supplement: Table S1 — Comparison of the B and T cell phenotypes in 8 month old B6.NZBc1c13 bicongenic mice with B6.NZBc1 and B6.NZBc13 congenic strains. Results are mean ± SD as determined by flow cytometry. Significance level for comparison of B6.NZBc1c13 mice with other mouse strains was determined by Mann-Whitney non-parametric test, *p<0.05, **p<0.005, ***p<0.0005. Numbers of 8 month old mice examined in each group are shown on the top unless otherwise indicated in brackets. Numbers shown in bold indicate significant difference p<0.05 from B6 control mice. (DOC) [file pone.0036761.s001.doc]

**Supplementary Table 1.** Comparison of the B and T cell phenotypes in 8 month old B6.NZBc1c13 bicongenic mice with B6.NZBc1 and B6.NZBc13 congenic strains.

|  | B6 | B6.NZBc1 | B6.NZBc13 | B6.NZBc1c13 | NZB |
| --- | --- | --- | --- | --- | --- |
|  | N = 18 | N = 28 | N = 9 | N = 31 | N = 9 |
| % B220^+^CD5^+^ | 1.46 ± 0.28 (9) | 1.79 ± 0.50 (10) | **3.49 ± 1.04^**^ (5)** | 1.54 ± 0.58 (18) | **2.41 ± 0.60^*^ (3)** |
| B220^+^ %CD21^low^CD23^–^ | 6.97 ± 2.33 | 8.14 ± 3.05 | **8.46 ± 2.59** | 7.69 ± 3.26 | **9.35 ± 2.63** |
| B220^+^ %CD21^int^CD23^+^ | 38.61 ± 10.09 | 40.49 ± 9.15 | 35.36 ± 8.03 | 34.15 ± 11.90 | **10.58 ± 6.06^***^** |
| B220^+^ %CD21^hi^CD23^+^ | 2.86 ± 1.75 | 2.60 ± 1.68 | 2.52 ± 1.60 | 2.34 ± 1.32 | **0.30 ± 0.38^***^** |
| B220^+^ %CD21^hi^CD23^-^ | 3.67 ± 1.22 | **2.36 ± 1.66** | **6.20 ± 2.96^***^** | **1.69 ± 0.81** | **1.58 ± 1.47** |
| B220^+^CD21^int^ % B7.1^+^ | 15.80 ± 12.14 (9) | **19.97 ± 4.58 (15)** | **24.42 ± 6.75 (5)** | **23.48 ± 12.30 (22)** | **30.51 ± 6.03 (5)** |
| B220^+^CD21^hi^ % B7.1^+^ | 28.93 ± 20.53 (9) | 29.11 ± 14.30 (15) | 45.22 ± 10.30 (5) | 33.09 ± 15.91 (22) | 36.06 ± 11.92 (5) |
| B220^+^CD21^int^ B7.2 MFI | 26.73 ± 16.14 (9) | **37.38 ± 10.89 (15)** | **42.84 ± 6.97 (5)** | **36.47 ± 12.63 (22)** | **53.57 ± 12.59 (5)** |
| B220^+^CD21^hi^ B7.2 MFI | 64.01 ± 58.87 (9) | 83.87 ± 50.13 (15) | 77.74 ± 17.72 (5) | **82.73 ± 52.19 (22)** | **91.98 ± 17.26 (5)** |
| B220^+^ %CD69^+^ | 6.06 ± 4.41 | **9.01 ± 2.90^**^** | **9.70 ± 4.25** | **14.62 ± 7.19** | 5.52 ± 2.19^***^ |
| CD4^+^ %CD69^+^ | 26.97 ± 6.61 | **51.04 ± 9.26** | **40.35 ± 3.95^**^** | **53.92 ± 9.23** | 36.15 ± 13.73^***^ |
| CD4^+^ %CD44^hi^CD62L^lo^ (Memory) | 38.48 ± 7.80 | **64.56 ± 15.35** | 51.52 ± 16.23 ^*^ | **66.03 ± 12.62** | **66.85 ± 14.00** |
| CD4^+^ %CD44^lo^CD62L^hi^ (Naïve) | 40.65 ± 10.71 | **12.85 ± 9.14** | **19.36 ± 11.26** | **12.08 ± 7.04** | **16.79 ± 11.96** |
