# Supplementary figures and images for: TLR Tolerance Reduces IFN-Alpha Production Despite Plasmacytoid Dendritic Cell Expansion and Anti-Nuclear Antibodies in NZB Bicongenic Mice
Source: PLoS One. 2012 May 4;7(5):e36761. doi: 10.1371/journal.pone.0036761 (PMC3344944; doi:10.1371/journal.pone.0036761)

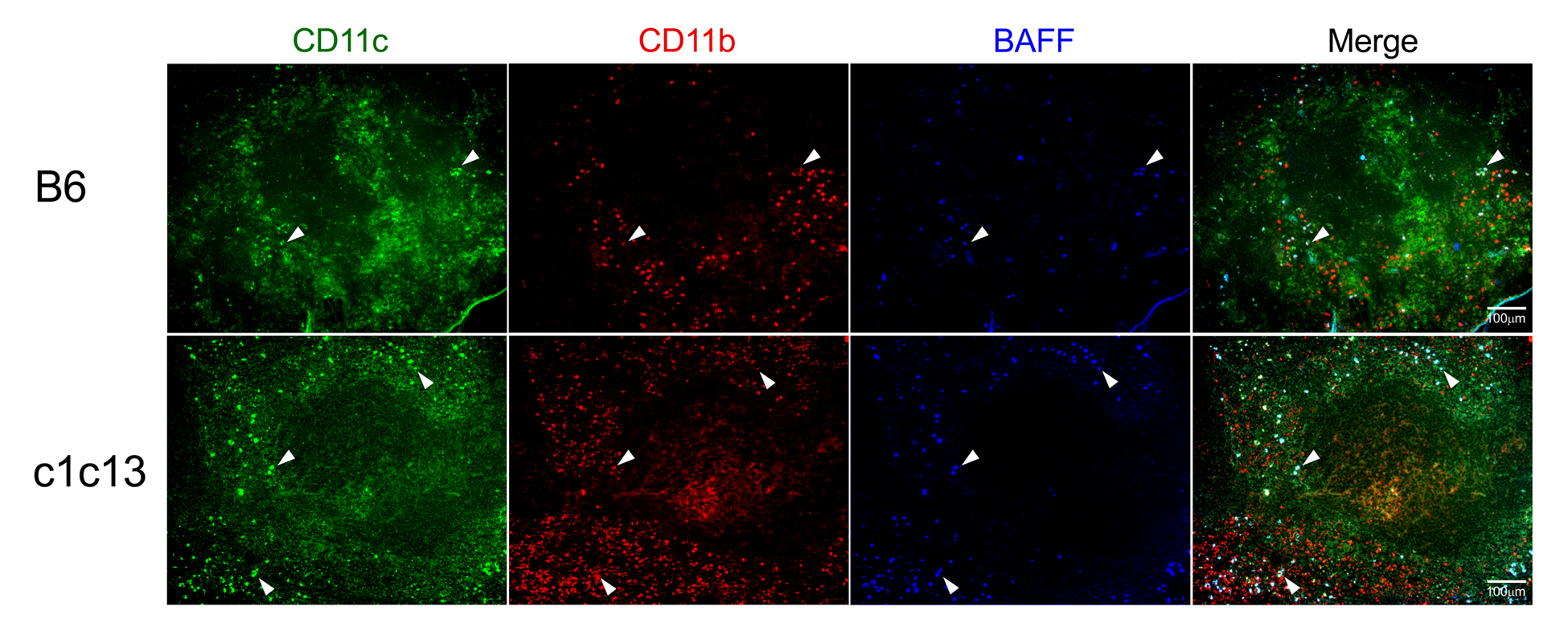

Supplement: Figure S1 — Splenic BAFF expression in B6 and c1c13 bicongenic mice. Cell populations producing BAFF were characterized by staining with biotinylated anti-CD11b, and FITC anti-CD11c with rabbit IgG anti-BAFF followed by AMCA-conjugated goat anti-rabbit IgG Ab. Biotin staining was revealed using rhodamine-conjugated streptavidin as a secondary reagent. Arrows indicate the same BAFF-producing CD11b+CD11c+ mDC in each image. The BAFF-producing CD11c+ cells did not stain with B220 (data not shown). Scale bar, 100 µm. (TIF) [file pone.0036761.s002.tif]

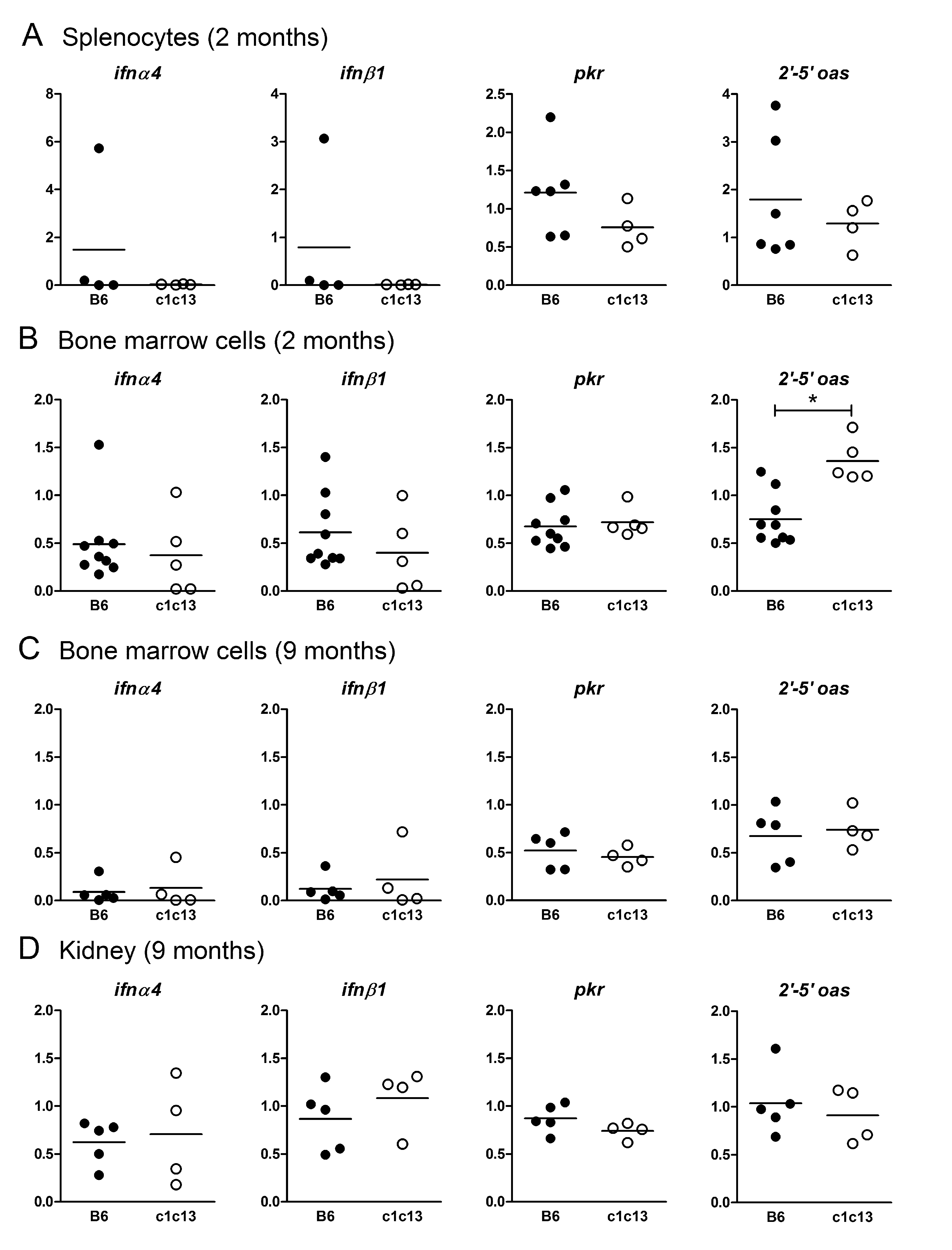

Supplement: Figure S2 — IFN-α/β and IFN-α-induced gene expression in various organs of 2 and 9 month-old B6 and bicongenic mice. Expression of IFN-α/β and IFN-α-induced (PKR and 2′-5′ OAS) genes was measured by qRT-PCR in B6 and B6.NZBc1c13 (c1c13) spleen (A) and bone marrow cells (B) at 2 months of age, and in the bone marrow cells (C) and kidneys (D) at 9 months of age. Relative mRNA expression of genes of interest normalized to β-actin mRNA expression. Each point represents the determination from an individual mouse. The p values for significant differences between B6.NZBc1c13 and B6 controls by Mann-Whitney non-parametric test were shown, *p<0.05. (TIF) [file pone.0036761.s003.tif]

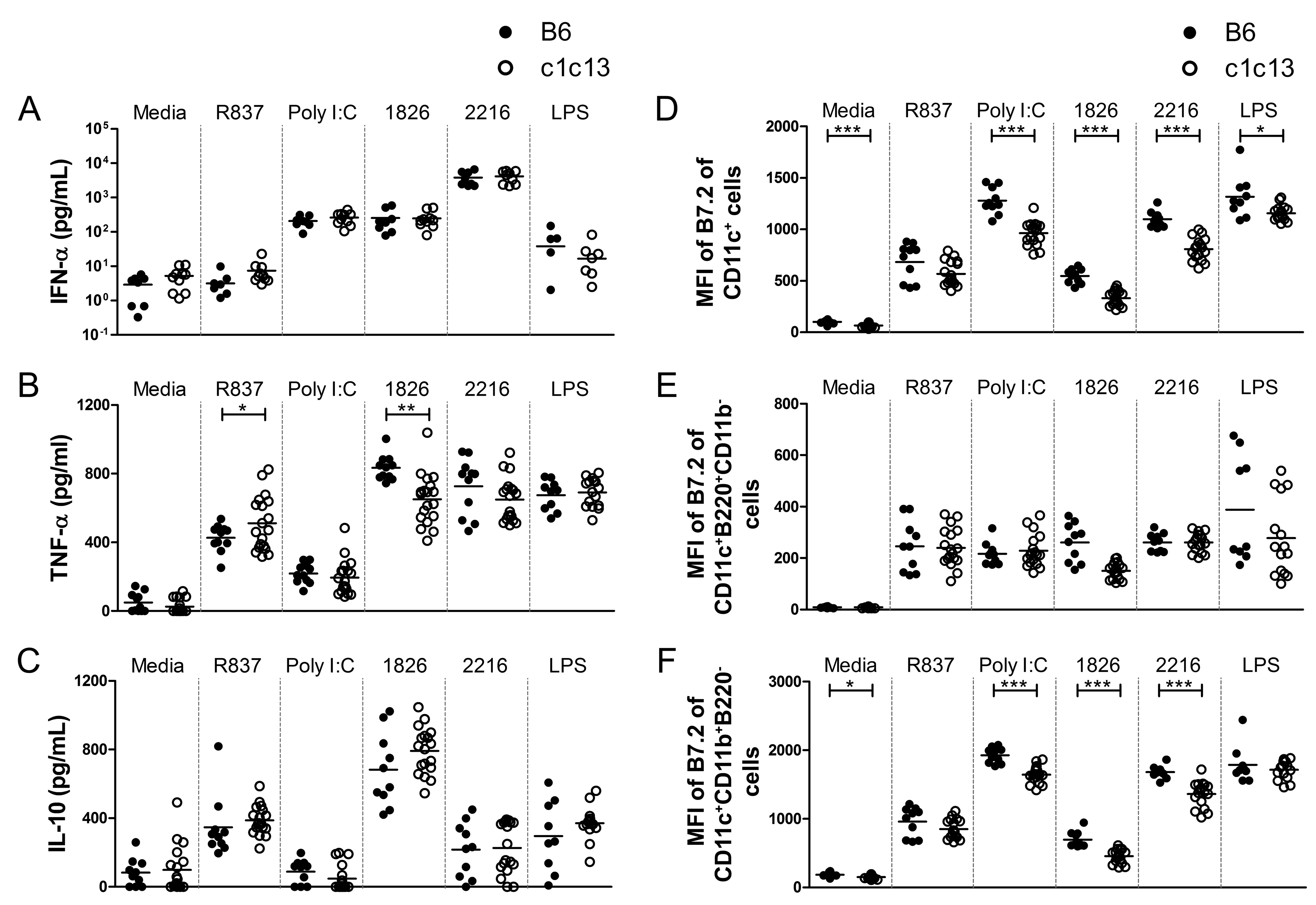

Supplement: Figure S3 — Similar levels of cytokine production, but reduced B7.2 expression in myeloid dendritic cells from bicongenic mice upon TLR stimulation. BMDC from 8–12 week-old mice were expanded in the presence of Flt3L for 7days and then cultured in the presence or absence of imiquimod R837, poly I:C, CpG 1826, CpG 2216 and LPS. (A) IFN-α, (B) TNF-α, and (C) IL-10 production in the culture supernatant was measured by ELISA. Each symbol represents the determination from an individual mouse, with background levels of cytokine with media alone subtracted. MFI for B7.2 expression on (D) CD11c+ total dendritic cells, (E) CD11c+B220+CD11b− plasmacytoid dendritic cells, and (F) CD11c+CD11b+B220− myeloid dendritic cells, as determined by flow cytometry. The p values for significant differences are shown, where *p<0.05, **p<0.005, ***p<0.0005, and were determined by the Mann-Whitney non-parametric test. Horizontal lines indicate the mean for each population examined. (TIF) [file pone.0036761.s004.tif]

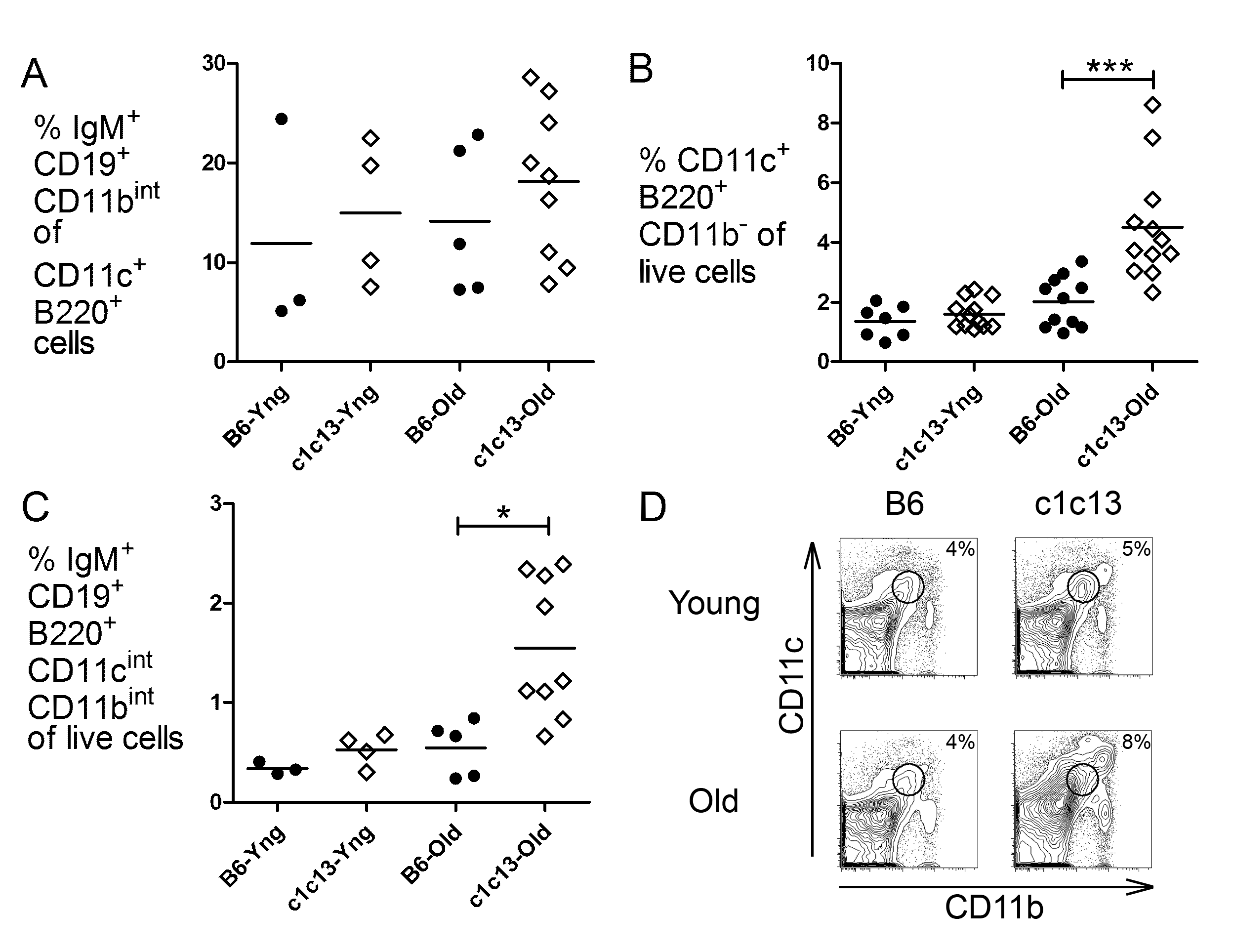

Supplement: Figure S4 — Examination of the splenic age-associated B cell population. (A) Similar proportion of contaminating IgM+ CD19+ CD11bint cells within the CD11c+ B220+ cell population across all strains and age groups. (B) Elevated levels of pDC in older bicongenic mice gated as CD11c+ B220+ CD11b− and excluding CD11bint ABC subset. (C) Increased proportion of ABCs is seen only in older bicongenic mice. ABCs were gated as IgM+ CD19+ B220+ CD11cint CD11bint and are expressed as a percentage of live cells. (D) Representative contour plots showing the gating and proportion of CD11cint CD11bint cells as a percentage of live cells in young and old B6 and bicongenic mice. The p values for significant differences are shown, where *p<0.05, ***p<0.0005, and were determined by the Mann-Whitney non-parametric test. (TIF) [file pone.0036761.s005.tif]
